# Reliability of the DSS-Swe Questionnaire

**DOI:** 10.1007/s11695-023-06841-7

**Published:** 2023-10-06

**Authors:** Anders Jans, Eva Rask, Johan Ottosson, Anders Magnuson, Eva Szabo, Erik Stenberg

**Affiliations:** 1 Department of Surgery, Faculty of Medicine and Health, Örebro University, 70185 Örebro, Sweden; 2 Faculty of Medicine and Health, University Health Care Research Centre, Örebro University, Örebro, Sweden; 3 Clinical Epidemiology and Biostatistics, School of Medical Sciences, Faculty of Medicine and Health, Örebro University, Örebro, Sweden

**Keywords:** Bariatric surgery, Hypoglycemia, Questionnaire, Translation, Reliability test

## Abstract

**Background:**

Symptomatic postbariatric hypoglycemia (PBH) is a known complication that can occur a few years after Roux-en-Y gastric bypass (RYGB). There is currently no established rating scale for PBH-associated symptoms developed for use in Swedish populations. The aim of the study was to translate an already existing questionnaire into Swedish and to test its reliability.

**Methods:**

The study included forward and backward translations of the original Dumping Severity Scale (DSS) questionnaire with 8 items regarding symptoms of early dumping and 6 items regarding hypoglycemia, with each item graded on a 4-point Likert scale. The reliability of the Swedish translated questionnaire (DSS-Swe) was estimated using internal consistency and test–retest methods.

**Results:**

A total of 200 patients were included in the study. Good internal consistency was demonstrated regarding the items related to early dumping symptoms, with a Cronbach’s alpha coefficient of 0.82, and very good agreement in terms of test–retest reliability, with an overall intraclass correlation coefficient (ICC) of 0.91 (95% CI 0.88–0.93). The items related to hypoglycemia yielded a good Cronbach’s alpha coefficient of 0.76 and an ICC of 0.89 (95% CI 0.85–0.91).

**Conclusion:**

The DSS-Swe questionnaire shows good reliability regarding both internal consistency and test–retest performance for use in Swedish populations.

**Graphical Abstract:**

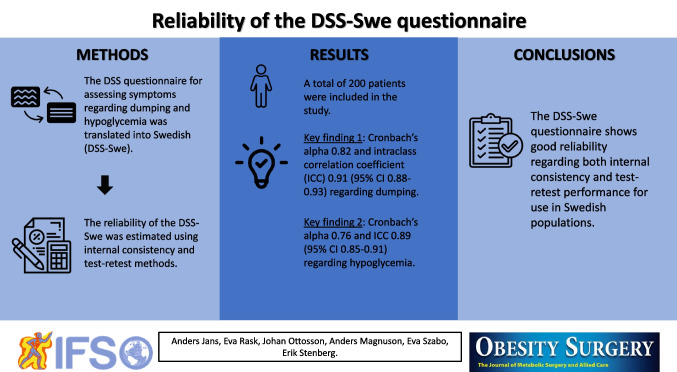

**Supplementary Information:**

The online version contains supplementary material available at 10.1007/s11695-023-06841-7.

## Introduction

The prevalence of obesity has risen significantly over the past 50 years, and in some countries, including Sweden, more than 50% of the population is overweight [1, 2]. It is well known that obesity is associated with diabetes and cardiovascular disease and increased overall mortality compared with normal-weight individuals [3, 4]. Bariatric surgery is currently the most effective treatment available to achieve long-term weight loss [5]. However, some patients suffer from postbariatric hypoglycemia (PBH). PBH is a hyperinsulinemic hypoglycemia with typical onset of symptoms after a median time between 2 and 3 years after surgery and rarely within the first postoperative year [6, 7]. The most common bariatric surgical methods at present are sleeve gastrectomy (SG) and Roux-en-Y gastric bypass (RYGB) [8]. While PBH generally has been considered a complication related to the Roux-en-Y construction, it has also been reported to be common after SG [9].

The incidence and prevalence of PBH after bariatric surgery are difficult to estimate since the definitions of PBH differ. Previous studies have estimated the 5-year incidence of suspected PBH after RYGB to range from less than 10% to more than 30% [6, 10, 11]. However, studies have shown that asymptomatic postprandial hypoglycemia is very common after bariatric surgery [12, 13]. Symptoms of PBH may include weakness, cognitive impairment, fatigue, dizziness, palpitations, tremor, sweating, paresthesia, and in the worst case, a life-threatening condition with convulsions and unconsciousness [10, 12, 14]. PBH usually occurs between 1 and 3 h after a meal [15]. Dumping syndrome, on the other hand, usually occurs within an hour after a meal and often causes gastrointestinal and vasomotor symptoms caused by osmotic effects, peptide hormone release, and autonomic neural responses [15].

The Dumping Severity Scale (DSS), a questionnaire that was initially constructed to measure the treatment response of octreotide on dumping syndrome, was developed by Arts and colleagues [16] and has recently also been used to assess symptoms of dumping and hypoglycemia after bariatric surgery [17, 18].

There is currently no available rating scale for PBH-associated symptoms developed for use in Swedish populations, and hence, there is a need for a questionnaire in Swedish for clinical follow-up of patients who have undergone bariatric surgery to identify symptoms of postbariatric hypoglycemia at an early stage.

The aim of this study was to translate the already existing DSS questionnaire into Swedish and to test its reliability for use in Swedish populations.

## Methods

The use of the original DSS questionnaire was authorized by Joris Arts.

This study consisted of two stages: translation of the DSS questionnaire and reliability testing of the translated questionnaire. The translated questionnaire was named DSS-Swe (Dumping Severity Score, Swedish version).

Arts’ “Dumping Severity Score” (DSS) [16] consists of a questionnaire with a 4-point Likert scale where the patient grades the intensity (scale, 0–3; 0, absent; 1, mild; 2, relevant; and 3, severe, interfering with daily activities) of 8 early dumping symptoms (sweating, flushing, dizziness, palpitations, abdominal pain, diarrhea, bloating, and nausea within 1 h after food ingestion) and 6 late dumping symptoms (sweating, palpitations, hunger, drowsiness/unconsciousness, tremor, and irritability starting more than 1 h after food ingestion).

### Translation

The first step was translation of the questionnaire, which was performed as follows:

1. Forward translation from English to Swedish was performed by three independent Swedish-speaking medical professionals.
2. The differences in the three Swedish translations were compared and discussed within the research group so that a preliminary Swedish version could be developed.
3. Four English-speaking translators translated the Swedish translation backward into English. The translators consisted of both health and nonhealth professionals.
4. The English translation was checked against the original English version to ensure that the versions were equivalent.
5. The first version of the Swedish questionnaire was tested on 10 patients who were then allowed to provide feedback regarding the comprehensibility of the questions. Some minor adjustments were made, but no major changes were considered necessary.

For the reliability test, all adult (≥ 18 years) patients presenting at Lindesberg Hospital, Sweden, during the inclusion period from September 2020 to April 2022 were considered for inclusion. Lindesberg Hospital is the center for bariatric surgery in Region Örebro County, Sweden. At the time of the preoperative visit or at a follow-up 6 months, 1 year, 2 years, or 5 years after bariatric surgery, patients were asked to complete the DSS-Swe questionnaire twice at 1–2-week intervals to allow a test–retest reliability analysis. The individuals had to have the ability to complete the DSS-Swe questionnaire on their own or with the support of an interpreter. The 1- to 2-week interval was chosen because it was long enough to prevent patients from remembering previous questionnaire answers, but it was not long enough to allow changes to the medical condition to any appreciable extent. Due to the COVID-19 pandemic, many follow-up visits were performed with telemedical assistance, limiting the number of patients available for inclusion. The number of patients included at each specific time point (preoperative visit or at a follow-up 6 months, 1 year, 2 years, or 5 years after bariatric surgery) was limited to 60 to reduce the imbalance of data.

Information on baseline characteristics, surgery, and follow-up were based on data from the Scandinavian Obesity Surgery Registry (SOReg). The SOReg was launched in 2007 as a national quality and research register reporting preoperative, intraoperative, and follow-up data at 30 days and 1, 2, 5, 10, and 15 years after surgery. At present, the registry covers virtually all metabolic surgical procedures in Sweden, with a very high acquisition rate and internal validity [19].

Based on previous studies with reliability tests of translated questionnaires, it was decided to include 200 patients [20, 21].

### Statistical Analysis

Data are presented as the number of individuals (*n*) with percentages of patients for categorical values, mean ± standard deviation (SD) for continuous variables assuming normal distribution, and median ± interquartile range (IQR) for continuous variables not assuming normal distribution.

The reliability was estimated using internal consistency and test–retest methods. Internal consistency was assessed using Cronbach’s alpha on the first of the two DSS-Swe questionnaires. The test–retest reliability of the sum score of the dumping and hypoglycemia sections of the questionnaire was determined using one-way random single measure intraclass correlation coefficient (ICC). Test–retest reliability per item was estimated using Cohen’s weighted kappa with quadratic weights.

ICC was assessed in accordance with a priori values: < 0.50 as poor, 0.50 to 0.75 as moderate, 0.75 to 0.90 as good, and > 0.90 as excellent.

The Wilcoxon signed ranks test was used to assess any systematic difference in the total score between questionnaires 1 and 2. A Bland‒Altman plot was used to describe the relation between the mean total score and the difference in total score per study participant.

Complete case analysis was used, i.e., when there were invalidly completed questionnaire items, that section of the questionnaire was excluded from further analysis.

SPSS Statistics version 27 (IBM, Armonk, New York, USA) was used for all statistical analyses.

### Ethics

The study was approved by the Ethics Committee in Stockholm (Dnr 2020–01257) and conducted in accordance with the ethical standards of the 1964 Helsinki Declaration and its later amendments. Informed consent was obtained from all individual participants included in the study.

## Results

**Fig. 1:**
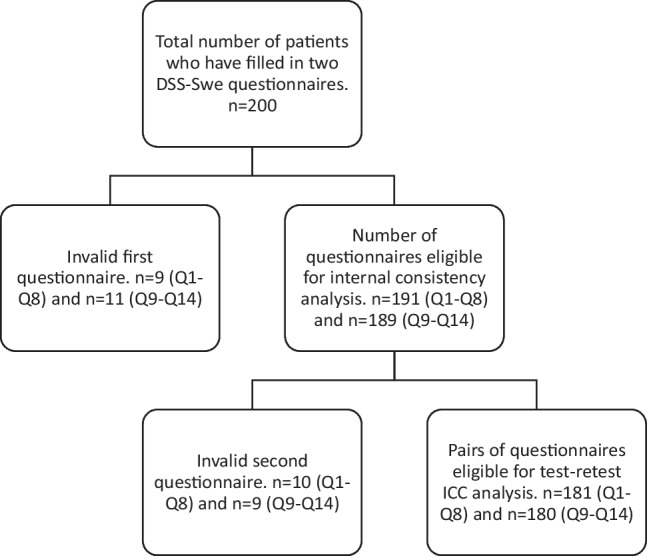
Study flowchart describing availability for analyses regarding first part (items Q1–Q8) and second part (items Q9–Q14) of the DSS-Swe

A total of 200 patients completed the DSS-Swe questionnaire twice. A study flowchart is presented in Fig. 1.

Average age at the inclusion in the study was 44.7 years (SD = 12.6), and the surgical methods consisted of gastric bypass 67.9% and sleeve gastrectomy 32.1% (Table 1). Baseline characteristics for each subgroup depending on the time point in relation to surgery are presented in Supplementary Table 1.

**Table 1:** Baseline characteristics

Characteristic	Missing data	
Individuals	0 (0.0%)	200 (100.0%)
Pre-operatively, *n* (%)		58 (29.0%)
6 months post-operatively, *n* (%)		18 (9.0%)
1 year post-operatively, *n* (%)		39 (19.5%)
2 years post-operatively, *n* (%)		52 (26.0%)
5 years post-operatively, *n* (%)		33 (16.5%)
Preoperative BMI, mean ± SD, kg/m^2^	0 (0.0%)	42.2 ± 6.2
Time between questionnaire 1 and 2, median (IQR), days	0 (0.0%)	9 (7–15)
Age, mean ± SD, years	0 (0.0%)	44.7 ± 12.6
Procedure, *n* (%)	4 (2.0%)^a^	
Gastric bypass, *n* (%)		133 (67.9%)
Sleeve gastrectomy, *n* (%)		63 (32.1%)
Sex	0 (0.0%)	
Female, *n* (%)		159 (79.5%)
Male, *n* (%)		41 (20.5%)
Comorbidity		
Sleep apnea, *n* (%)	0 (0.0%)	39 (19.5%)
Hypertension, *n* (%)	0 (0.0%)	51 (25.5%)
Dyslipidemia, *n* (%)	0 (0.0%)	15 (7.5%)
Dyspepsia/gastroesophageal reflux disease, *n* (%)	0 (0.0%)	24 (12.0%)
Depression, *n* (%)	0 (0.0%)	20 (10.0%)
Previous pulmonary embolus/deep venous thrombosis, *n* (%)	0 (0.0%)	4 (2.0%)
Type 2 diabetes mellitus prior to surgery, *n* (%)	0 (0.0%)	31 (15.5%)
Glycosylated hemoglobin A1c pre-operatively, mmol/mol, mean ± SD	1 (0.5%)	40.0 ± 9.7
Education	12 (6.0%)	
Primary education ≤ 9 yrs, *n* (%)		13 (6.9%)
Secondary education 10–12 yrs, *n* (%)		135 (71.8%)
Higher education, *n* (%)		40 (21.3%)

*BMI* = body mass index; *SD* = standard deviation; *IQR* = interquartile range

^a^Including patients not operated yet

The translated questionnaire items in relation to those in the original English-language version are shown in Table 2.

**Table 2:** Symptoms in original DSS and the translated DSS-Swe with Cronbach's alpha internal consistency calculation

Item	DSS	DSS-Swe	Cronbach's alpha if item deleted
Overall Cronbach’s alpha for items Q1–Q8 = 0.82			
	Symptoms within 1 h after a meal (dumping)		
Q1	Sweating	Svettning	0.79
Q2	Flushing	Värmevallning/häftig rodnad	0.80
Q3	Dizziness	Yrsel	0.79
Q4	Palpitations	Hjärtklappning	0.79
Q5	Abdominal pain	Magsmärta	0.79
Q6	Diarrhea	Diarré	0.81
Q7	Bloating	Uppblåsthet	0.80
Q8	Nausea	Illamående	0.80
Overall Cronbach’s alpha for items Q9–Q14 = 0.76			
	Symptoms 1 to 3 h after a meal (hypoglycemia)		
Q9	Sweating	Svettning	0.71
Q10	Palpitations	Hjärtklappning	0.73
Q11	Hunger	Hunger	0.80
Q12	Drowsiness/unconsciousness	Dåsighet/medvetslöshet	0.72
Q13	Tremor	Skakningar/darrningar	0.69
Q14	Irritability	Blir lättretlig	0.72

Analysis of internal consistency, reflecting to which extent items in the questionnaire measure various aspects of the same characteristic, showed an overall Cronbach’s alpha of 0.82 for Q1–Q8 (early dumping symptoms). Corresponding analysis for Q9–Q14 (late hypoglycemic symptoms) yielded a Cronbach’s alpha of 0.76 (Table 2). In a subgroup analysis stratified by pre- and postoperative patients, good internal consistency was demonstrated for both groups regarding Q1–Q8 (Cronbach’s alpha of 0.82 and 0.81, respectively), while the preoperative group had slightly lower internal consistency compared to the postoperative group regarding Q9–Q14 (Supplementary Table 2). The test–retest reliability, regarding the total score from Q1–Q8 yielded an overall ICC of 0.91, and for Q9–Q14, an overall ICC of 0.89 (Table 3). Test–retest analysis with Cohen’s weighted kappa for each individual item gave values between 0.75 and 0.89 with *p* < 0.001 for each item (Table 4).

**Table 3:** Test–retest reliability for sum scores Q1–Q8 and Q9–Q14

Item	Patient group	Missing data	Median sum score (IQR)	ICC (95% CI)
Q1–Q8	All patients	19 (9.5%)	3 (1–6)	0.91 (0.88–0.93)
	Pre-operative	5 (8.6%)	2 (0–5)	0.79 (0.66–0.87)
	Post-operative	14 (9.9%)	3 (1–6.75)	0.94 (0.92–0.96)
Q9–Q14	All patients	20 (10.0%)	1 (0–3)	0.89 (0.85–0.91)
	Pre-operative	8 (13.8%)	1 (0–2)	0.67 (0.49–0.80)
	Post-operative	12 (8.5%)	2 (0–3.75)	0.92 (0.89–0.94)

*CI* = confidence Interval; *ICC* = intraclass correlation coefficient; *IQR* = interquartile range

**Table 4:** Test–retest reliability per item

Item	Missing data, *n* (%)	Cohen’s weighted kappa (95% CI)
Q1	8 (4.0%)	0.77 (0.66–0.89)
Q2	3 (1,5%)	0.85 (0.77–0.93)
Q3	6 (3.0%)	0.83 (0.75–0.91)
Q4	8 (4.0%)	0.89 (0.84–0.94)
Q5	5 (2.5%)	0.85 (0.79–0.91)
Q6	6 (3.0%)	0.86 (0.79–0.92)
Q7	2 (1.0%)	0.75 (0.66–0.85)
Q8	6 (3.0%)	0.79 (0.68–0.89)
Q9	10 (5.0%)	0.81 (0.71–0.92)
Q10	10 (5.0%)	0.75 (0.59–0.92)
Q11	13 (6.5%)	0.79 (0.69–0.88)
Q12	10 (5.0%)	0.88 (0.80–0.95)
Q13	8 (4.0%)	0.86 (0.78–0.94)
Q14	10 (5.0%)	0.89 (0.83–0.96)

CI = Confidence Interval

**Fig. 2:**
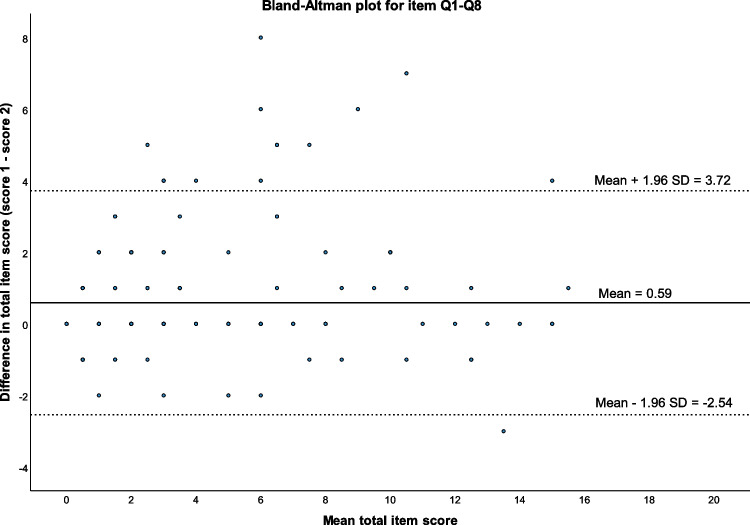
Bland–Altman plot describing relation between mean total and difference in total score for items 1–8 per study participant

When comparing the total score between the two sampling occasions, a higher total score was seen for early dumping symptoms in questionnaire 1 compared with questionnaire 2 (mean difference 0.59, 95% CI 0.36–0.83, *p* < 0.001). The difference was observed both during preoperative assessment (mean difference for items Q1–Q8 0.92, 95% CI 0.34–1.51, *p* = 0.005) and after surgery (mean difference for items Q1–Q8 0.45, 95% CI 0.23–0.68, *p* < 0.001). No statistically significant differences between occasions were seen for items Q9–Q14. The differences are illustrated with Bland‒Altman plots (Fig. 2 and Supplementary Fig. 1).

Item Q11 (hunger) impairs the internal consistency for the second part of the DSS-Swe (Q9–Q14) with items related to hypoglycemia (Cronbach’s alpha 0.76 with Q11, and 0.80 when excluding Q11, Table 2). In a subgroup analysis, this effect concerning Q11 was seen in the postoperative group, but not in the preoperative group.

## Discussion

The DSS-Swe has good internal consistency and test–retest performance for both dumping-related symptoms and hypoglycemia in a Swedish population before and after bariatric surgery.

An interesting finding is that Item Q11 (hunger) reduced the internal consistency for hypoglycemia-related items (Q9–Q14) in the postoperative setting. However, this does not seem entirely unreasonable, as “hunger” is a symptom not specifically associated with hypoglycemia to the same extent as other symptoms in that part of the questionnaire.

There are several different questionnaires for estimating hypoglycemia symptoms, such as the DSS, the Edinburgh Hypoglycemia Scale, and the Sigstad Dumping Score, but none of these questionnaires has been validated for the assessment of postbariatric hypoglycemia, and most of them do not differentiate between early dumping symptoms and PBH [12, 22]. The major benefit of Arts’ DSS questionnaire, and the reason it was chosen for translation and reliability testing, is the relative conciseness and clarity of the questionnaire, making it suitable for clinical use. Since the DSS divides the symptoms between early dumping and later hypoglycemia, it can be considered particularly suitable for the follow-up of patients who have undergone bariatric surgery.

The original DSS was created to provide an index of severity by quantifying symptoms, but the diagnostic performance has not been addressed [15]. The questionnaire has never been formally validated, but it has been found that the symptoms of drowsiness and the total dumping severity score have a borderline significant correlation with nadir plasma glucose [16, 22].

When diagnosing symptomatic hypoglycemia, Whipple’s triad should usually be confirmed, including the presence of typical hypoglycemia symptoms, confirmed low blood/interstitial glucose, and symptom regression when the glucose value normalizes [23]. Unfortunately, there are currently no established diagnostic criteria for PBH based on specific plasma glucose levels. However, the American Society for Metabolic and Bariatric Surgery (ASMBS) suggests that PBH should be considered when the time point of symptom onset is more than 1 year after bariatric surgery, fasting plasma glucose and fasting insulin levels are normal, provocation test results are positive, and there is a correlation of symptoms with hypoglycemia followed by spontaneous resolution of hypoglycemia [12].

PBH affects a relatively large proportion of patients who have undergone bariatric surgery, particularly those with risk factors such as low preoperative HbA1c, female sex, RYGB (compared with SG), and large postoperative weight loss [6, 10, 11]. Moreover, bariatric surgery has become increasingly common, with more than 500,000 bariatric surgeries performed annually worldwide [8]. Consequently, PBH has the potential to become a growing clinical problem. The questionnaire is therefore likely to be valuable as part of postoperative follow-up to identify patients with symptoms consistent with symptomatic PBH. However, it is important to remember that a questionnaire such as the DSS-Swe should only be used as a screening tool. Before diagnosis and possible treatment for PBH can be carried out, measurement of plasma/interstitial glucose at the time of symptoms should be performed.

The reliability of a questionnaire can be affected by how the translation to another language is carried out and how the questions are perceived. However, Arts’ DSS questionnaire is one of the most used questionnaires internationally when assessing the occurrence of symptoms related to dumping and hypoglycemia after bariatric surgery, and we are not aware that the questionnaire has been reliability tested in its English language version. Our reliability test of the Swedish version of the DSS can hopefully contribute to increased knowledge regarding the original English-language version including how the various items contribute to the internal consistency of the entire questionnaire.

### Limitations

Since the start of the study, all patients considered for bariatric surgery or planned for postoperative follow-up were offered the opportunity to participate in the study, but registration of those who declined participation has not been recorded. It has therefore not been possible to perform a sensitivity analysis of patients who declined participation. However, the characteristics of patients in the present study are similar to those of the bariatric surgical population in Sweden [24]. When calculating internal consistency with Cronbach’s alpha, the first of the two completed questionnaires was used. While different strategies could have been used, the reason why only the first questionnaire was used for internal consistency analysis was that this approach best represents the scenario in which patients in clinical practice only complete a single questionnaire.

In the test–retest reliability test, a 1–2-week interval between the questionnaires was intended to allow a sufficiently long time so that the participants would not clearly remember their answers from the previous questionnaire but also a sufficiently short time so that the medical condition would not have changed. A small proportion of patients did not keep this time span. Although this was a violation of the intended plan, the small proportion of patients, in combination with the unlikely change in hypoglycemia over a short period of time, suggests that this violation is unlikely to influence the overall results. Finally, the absence of a generally accepted definition of PBH made a formal validation of DSS-Swe impossible.

## Conclusion

The DSS-Swe questionnaire shows good reliability regarding both internal consistency and test–retest performance for use in Swedish populations.

### Supplementary Information

Below is the link to the electronic supplementary material.

Supplementary file1 (DOCX 19 KB)

Supplementary file2 (DOCX 14 KB)

Supplementary file3 (EPS 6727 KB)

## Data Availability

The data that support the findings of this study are available from the corresponding author upon reasonable request.
