# Supplementary material for: Reliability of the DSS-Swe Questionnaire
Source: Obes Surg. 2023 Oct 6;33(11):3487–93. doi: 10.1007/s11695-023-06841-7 (PMC10603007; doi:10.1007/s11695-023-06841-7)
Supplement: Supplementary file 1 — Supplementary file1 (DOCX 19 KB) [file 11695_2023_6841_MOESM1_ESM.docx]

| **Supplementary table 1. Baseline characteristics** | **All patients (n=200)** | | **Pre-operatively (n=58)** | | **All post-operatively (n=142)** | | **6 months post-op (n=18)** | | **1 year post-op (n=39)** | | **2 years post-op (n=52)** | | **5 years post-op (n=33)** | | **P value^b^** |
| --- | --- | --- | --- | --- | --- | --- | --- | --- | --- | --- | --- | --- | --- | --- | --- |
| **Characteristic** | **Missing data** |  | **Missing data** |  | **Missing data** |  | **Missing data** |  | **Missing data** |  | **Missing data** |  | **Missing data** |  |  |
| Preoperative BMI, mean ± SD, kg/m^2^ | 0 (0.0%) | 42.2 ± 6.2 | 0 (0.0%) | 42.0 ± 5.4 | 0 (0.0%) | 42.2 ± 6.5 | 0 (0.0%) | 43.5 ± 7.3 | 0 (0.0%) | 41.9 ± 7.2 | 0 (0.0%) | 42.1 ± 6.7 | 0 (0.0%) | 42.1 ± 5.0 | 0.84 |
| Time between questionnaire 1 and 2, median (IQR), days | 0 (0.0%) | 9 (7-15) | 0 (0.0%) | 15 (8-20) | 0 (0.0%) | 9 (7-13) | 0 (0.0%) | 9 (7-14) | 0 (0.0%) | 8 (6-12) | 0 (0.0%) | 9 (7-13) | 0 (0.0%) | 9 (7-13) | <0.001 |
| Age, mean ± SD, years | 0 (0.0%) | 44.7 ± 12.6 | 0 (0.0%) | 39.4 ± 11.1 | 0 (0.0%) | 46.9 ± 12.6 | 0 (0.0%) | 50.2 ± 10.6 | 0 (0.0%) | 43.5 ± 11.4 | 0 (0.0%) | 46.0 ± 11.7 | 0 (0.0%) | 50.5 ± 15.0 | <0.001 |
| Procedure, n (%) | 4 (2.0%)^a^ |  | 4 (6.9%)^a^ |  | 0 (0.0%) |  | 0 (0.0%) |  | 0 (0.0%) |  | 0 (0.0%) |  | 0 (0.0%) |  | 0.42 |
| Gastric bypass, n (%) |  | 133 (67.9%) |  | 39 (72.2%) |  | 94 (66.2%) |  | 13 (72.2%) |  | 23 (59.0%) |  | 34 (65.4%) |  | 24 (72.7%) |  |
| Sleeve gastrectomy, n (%) |  | 63 (32.1%) |  | 15 (27.8%) |  | 48 (33.8%) |  | 5 (27.8%) |  | 16 (41.0%) |  | 18 (34.6%) |  | 9 (27.3%) |  |
| Sex | 0 (0.0%) |  | 0 (0.0%) |  | 0 (0.0%) |  | 0 (0.0%) |  | 0 (0.0%) |  | 0 (0.0%) |  | 0 (0.0%) |  | 0.13 |
| Female, n (%) |  | 159 (79.5%) |  | 50 (86.2%) |  | 109 (76.8%) |  | 14 (77.8%) |  | 30 (76.9%) |  | 39 (75.0%) |  | 26 (78.8%) |  |
| Male, n (%) |  | 41 (20.5%) |  | 8 (13.8%) |  | 33 (23.3%) |  | 4 (22.2%) |  | 9 (23.1%) |  | 13 (25.0%) |  | 7 (21.2%) |  |
| Comorbidity prior to surgery |  |  |  |  |  |  |  |  |  |  |  |  |  |  |  |
| Sleep apnoea, n (%) | 0 (0.0%) | 39 (19.5%) | 0 (0.0%) | 10 (17.2%) | 0 (0.0%) | 29 (20.4%) | 0 (0.0%) | 7 (38.9%) | 0 (0.0%) | 8 (20.5%) | 0 (0.0%) | 9 (17.6%) | 0 (0.0%) | 5 (15.2%) | 0.61 |
| Hypertension, n (%) | 0 (0.0%) | 51 (25.5%) | 0 (0.0%) | 9 (15.5%) | 0 (0.0%) | 42 (29.6%) | 0 (0.0%) | 7 (38.9%) | 0 (0.0%) | 11 (28.2%) | 0 (0.0%) | 15 (28.8%) | 0 (0.0%) | 9 (27.3%) | 0.038 |
| Dyslipidaemia, n (%) | 0 (0.0%) | 15 (7.5%) | 0 (0.0%) | 2 (3.4%) | 0 (0.0%) | 13 (9.2%) | 0 (0.0%) | 3 (16.7%) | 0 (0.0%) | 2 (5.1%) | 0 (0.0%) | 3 (5.8%) | 0 (0.0%) | 5 (15.2%) | 0.16 |
| Dyspepsia / Gastroesophageal reflux disease, n (%) | 0 (0.0%) | 24 (12.0%) | 0 (0.0%) | 9 (15.5%) | 0 (0.0%) | 15 (10.6%) | 0 (0.0%) | 2 (11.1%) | 0 (0.0%) | 5 (12.8%) | 0 (0.0%) | 4 (7.7%) | 0 (0.0%) | 4 (12.1%) | 0.33 |
| Depression, n (%) | 0 (0.0%) | 20 (10.0%) | 0 (0.0%) | 10 (17.2%) | 0 (0.0%) | 10 (7.0%) | 0 (0.0%) | 1 (5.6%) | 0 (0.0%) | 6 (15.4%) | 0 (0.0%) | 3 (5.8%) | 0 (0.0%) | 0 (0.0%) | 0.29 |
| Previous pulmonary embolus / Deep venous thrombosis, n (%) | 0 (0.0%) | 4 (2.0%) | 0 (0.0%) | 2 (3.4%) | 0 (0.0%) | 2 (1.4%) | 0 (0.0%) | 0 (0.0%) | 0 (0.0%) | 0 (0.0%) | 0 (0.0%) | 1 (1.9%) | 0 (0.0%) | 1 (3.0%) | 0.35 |
| Type 2 diabetes mellitus prior to surgery, n (%) | 0 (0.0%) | 31 (15.5%) | 0 (0.0%) | 4 (6.9%) | 0 (0.0%) | 27 (19.0%) | 0 (0.0%) | 7 (38.9%) | 0 (0.0%) | 8 (20.5%) | 0 (0.0%) | 7 (13.5%) | 0 (0.0%) | 5 (15.2%) | 0.032 |
| Glycosylated Haemoglobin A1c pre-operatively, mmol/mol, mean ± SD | 1 (0.5%) | 40.0 ± 9.7 | 0 (0.0%) | 38.0 ± 4.2 | 1 (0,7%) | 40.8 ± 11.1 | 0 (0.0%) | 44.4 ± 13.1 | 1 (2.6%) | 42.0 ± 12.4 | 0 (0.0%) | 40.0 ± 10.2 | 0 (0.0%) | 38.8 ± 9.5 | 0.063 |
| Education | 12 (6.0%) |  | 7 (12.1%) |  | 5 (3.5%) |  | 1 (5.6%) |  | 1 (2.6%) |  | 0 (0.0%) |  | 3 (9.1%) |  | 0.52 |
| Primary education ≤9 yrs, n (%) |  | 13 (6.9%) |  | 4 (7.8%) |  | 9 (6.6%) |  | 3 (17.6%) |  | 3 (7.9%) |  | 2 (3.8%) |  | 1 (3.3%) |  |
| Secondary education 10-12 yrs, n (%) |  | 135 (71.8%) |  | 39 (76.5%) |  | 96 (70.1%) |  | 10 (58.8%) |  | 31 (81.6%) |  | 36 (69.2%) |  | 19 (63.3%) |  |
| Higher education, n (%) |  | 40 (21.3%) |  | 8 (15.7%) |  | 32 (23.4%) |  | 4 (23.5%) |  | 4 (10.5%) |  | 14 (26.9%) |  | 10 (33.3%) |  |
| BMI = body mass index; SD = standard deviation; IQR = interquartile range. | |  |  |  |  |  |  |  |  |  |  |  |  |  |  |
| ^a^ Including patients not operated yet. |  |  |  |  |  |  |  |  |  |  |  |  |  |  |  |
| ^b^ P value for comparison between "pre-operatively" and "all post-operatively" groups. T-test was used for continuous variables with assumed normal distribution and Mann-Whitney U test for assumed non-normal distributed continuous variables. Chi-Square was used for categorical variables. | | | | | | | | | | | | | | | |
