# Supplementary material for: Reliability of the DSS-Swe Questionnaire
Source: Obes Surg. 2023 Oct 6;33(11):3487–93. doi: 10.1007/s11695-023-06841-7 (PMC10603007; doi:10.1007/s11695-023-06841-7)
Supplement: Supplementary file 2 — Supplementary file2 (DOCX 14 KB) [file 11695_2023_6841_MOESM2_ESM.docx]

| **Supplementary Table 2. Symptoms in original DSS and the translated DSS-Swe with Cronbach's alpha internal consistency calculation.** | | | | | |
| --- | --- | --- | --- | --- | --- |
| **Item** | **DSS** | **DSS-Swe** | **Cronbach's alpha if item deleted (all patients)** | **Cronbach's alpha if item deleted (pre-operative patients)** | **Cronbach's alpha if item deleted (post-operative patients)** |
|  | **Symptoms within 1 hour after a meal (dumping)** | |  |  |  |
| Q1 | Sweating | Svettning | 0.79 | 0.81 | 0.79 |
| Q2 | Flushing | Värmevallning/häftig rodnad | 0.80 | 0.81 | 0.80 |
| Q3 | Dizziness | Yrsel | 0.79 | 0.83 | 0.78 |
| Q4 | Palpitations | Hjärtklappning | 0.79 | 0.83 | 0.79 |
| Q5 | Abdominal pain | Magsmärta | 0.79 | 0.81 | 0.79 |
| Q6 | Diarrhea | Diarré | 0.81 | 0.84 | 0.80 |
| Q7 | Bloating | Uppblåsthet | 0.80 | 0.82 | 0.79 |
| Q8 | Nausea | Illamående | 0.80 | 0.83 | 0.79 |
|  |  |  |  |  |  |
| Overall Cronbach's alpha for Q1-Q8 = 0.82 (all patients), 0.84 (pre-operative patients), 0.81 (post-operative patients) | | | | |  |
|  |  |  |  |  |  |
|  | **Symptoms 1 to 3 hours after a meal (hypoglycemia)** | |  |  |  |
| Q9 | Sweating | Svettning | 0.71 | 0.60 | 0.72 |
| Q10 | Palpitations | Hjärtklappning | 0.73 | 0.62 | 0.74 |
| Q11 | Hunger | Hunger | 0.80 | 0.63 | 0.81 |
| Q12 | Drowsiness/  unconsciousness | Dåsighet/medvetslöshet | 0.72 | 0.64 | 0.73 |
| Q13 | Tremor | Skakningar/darrningar | 0.69 | 0.50 | 0.69 |
| Q14 | Irritability | Blir lättretlig | 0.72 | 0.47 | 0.75 |
|  |  |  |  |  |  |
| Overall Cronbach's alpha for Q9-Q14 = 0.76 (all patients), 0.65 (pre-operative patients), 0.78 (post-operative patients) | | | | |  |
